# Genome-wide discovery of di-nucleotide SSR markers based on whole genome re-sequencing data of *Cicer arietinum* L. and *Cicer reticulatum* Ladiz

**DOI:** 10.1038/s41598-023-37268-w

**Published:** 2023-06-26

**Authors:** Duygu Sari, Hatice Sari, Cengiz Ikten, Cengiz Toker

**Affiliations:** 1 Department of Field Crops, Faculty of Agriculture, Akdeniz University, 07070 Antalya, Turkey; 2 Department of Plant Protection, Faculty of Agriculture, Akdeniz University, 07070 Antalya, Turkey

**Keywords:** Plant molecular biology, Plant sciences, Genomic analysis, Sequencing, Software

## Abstract

Simple sequence repeats (SSRs) are valuable genetic markers due to their co-dominant inheritance, multi-allelic and reproducible nature. They have been largely used for exploiting genetic architecture of plant germplasms, phylogenetic analysis, and mapping studies. Among the SSRs, di-nucleotide repeats are the most frequent of the simple repeats distributed throughout the plant genomes. In present study, we aimed to discover and develop di-nucleotide SSR markers by using the whole genome re-sequencing (WGRS) data from *Cicer arietinum* L. and *C. reticulatum* Ladiz. A total of 35,329 InDels were obtained in *C. arietinum,* whereas 44,331 InDels in *C. reticulatum*. 3387 InDels with 2 bp length were detected in *C. arietinum*, there were 4704 in *C. reticulatum*. Among 8091 InDels, 58 di-nucleotide regions that were polymorphic between two species were selected and used for validation. We tested primers for evaluation of genetic diversity in 30 chickpea genotypes including *C. arietinum*, *C. reticulatum, C. echinospermum* P.H. Davis, *C. anatolicum* Alef., *C. canariense* A. Santos & G.P. Lewis, *C. microphyllum* Benth., *C. multijugum* Maesen, *C. oxyodon* Boiss. & Hohen. and *C. songaricum* Steph ex DC. A total of 244 alleles were obtained for 58 SSR markers giving an average of 2.36 alleles per locus. The observed heterozygosity was 0.08 while the expected heterozygosity was 0.345. Polymorphism information content was found to be 0.73 across all loci. Phylogenetic tree and principal coordinate analysis clearly divided the accessions into four groups. The SSR markers were also evaluated in 30 genotypes of a RIL population obtained from an interspecific cross between *C. arietinum* and *C. reticulatum.* Chi-square (χ^2^) test revealed an expected 1:1 segregation ratio in the population. These results demonstrated the success of SSR identification and marker development for chickpea with the use of WGRS data. The newly developed 58 SSR markers are expected to be useful for chickpea breeders.

## Introduction

Chickpea (*Cicer arietinum* L.) is one of the valuable cool-season grain legume crops in the world. It is a self-pollinated and diploid plant (2n = 2x = 16) with a genome size of ~ 740 Mb^1^ which is considerably less than other important legume crops like pea, lentil, alfalfa, soybean and peanut^2^. The genus *Cicer* L. belongs to the family Fabaceae, subfamily Faboideae and contains a total of 49 taxa with 9 annuals and 40 perennials^3–6^. Toker et al.^7^ has been recently introduced a new annual wild *Cicer* species, thereby increasing the count to 10 annual species. *C. arietinum* is solely cultivated species of the genus. *C. reticulatum* is considered to be the wild progenitor of the cultivated chickpea^8^. It is crossable with the cultivated chickpea and possesses 2n = 2x = 16 chromosommes with a smaller genome size of 416 Mb than that of the cultivated chickpea^9^.

Chickpea plays valuable roles in human diet as a rich source of dietary proteins, complex carbohydrates and micronutrients such as iron, potassium and zinc as well as vitamins A and B in addition to folate and thiamine^10^. Because of its capacity of biological fixation of atmospheric nitrogen through nodulation with *Rhizobium* species, it is an advantageous crop in crop rotation^11^. Also, chickpea is the most important cool season food legume in the arid and semi-arid areas under rainfed conditions^12^. Globally, harvested area was approximately 14.8 million ha and total production was almost 15.1 million tons of chickpeas in 2020^13^. It is widely grown and consumed in India, Pakistan, Iran and Turkey^13^.

Various biotic and abiotic factors have been affecting the chickpea production in the worldwide^14,15^. Due to limited genetic diversity in cultivated chickpea, it has been restricted achievement in respect to efforts for increasing the productivity^16^. Conventional methods have been used in crop breeding and tolerance to the environmental stresses while molecular breeding approaches have potential to accelerate the process of developing new cultivars. Also, the effective usage of plant genetic resources in breeding might be possible with the awareness and information of genetic variation present within individuals or populations.

Molecular markers explore the genetic diversity at the DNA level and have the capability to reflect the precise genetic diversity between genotypes^17^. In chickpea, random amplified polymorphic DNA (RAPD)^18–20^, amplified fragment length polymorphism (AFLP)^21,22^, simple sequence repeat (SSR)^23^, inter simple sequence repeat (ISSR)^24–26^ and internal transcribed spacer (ITS)^27^ have been used for genetic diversity analysis in different germplasm. Recently, an extensive development has been made regarding the improvement of several genomic or transcript-based SSR markers and SNP markers and their deployment in the large-scale genomics and breeding programs in chickpea^28–35^. In contrast to SNP markers, SSRs are very convenient and easy to use. SSRs can be found in both coding and noncoding regions of all higher organisms. The genome wide occurrence, co-dominant inheritance, highly polymorphic and multi-allelic nature promote wide utilization of SSRs^36–38^. Earlier, the usual protocol for isolation microsatellite sequences was utilization of microsatellite-enriched libraries by cloning and Sanger sequencing method, which was costly, difficult, and time consuming^39^.

Recently, development of next-generation sequencing (NGS) technologies has prompted the fast and cost-effective SSR discovery in many crops. There are now numerous methods that apply NGS for genotyping, reduced representation libraries (RRLs), restriction-site-associated DNA sequencing (RADseq), genotyping-by-sequencing (GBS), whole-genome resequencing (WGRS)^40–42^. WGRS is more appropriate for pre-breeding activities where less number of elite parents, landraces and wild species require to be examined delicately for genome variation (SNPs, CNV, structural variation) and association studies^43^. Efficiency of WGRS have been shown in many such crops such as rice^44,45^, sorghum^46^, cotton^47^, soybean^48^, tomato^49^, and chickpea^50–53^. In view of above prospects, genome-wide SSR markers were developed in chickpea in the present study. The utility of these developed markers in F_6_ population derived from an interspecific cross between *C. arietinum* and *C. reticulatum* was accessed. The cross-transferability of these markers was also examined across 30 chickpea genotypes including cultivated and wild types.

## Results

### Genotyping

A total of 2.01 GB and 2.16 GB raw sequence reads of *C. arietinum* and *C. reticulatum* were generated from 150 bp paired-end sequencing. *C. arietinum* had 34.77 M reads and 33% guanine-cytosine (GC) content while *C. reticulatum* had 33.60 M reads and 34% GC content. The means of reads mapped to the *C. arietinum* reference genome were 97.56% and 96.62% in *C. arietinum* and *C. reticulatum*, respectively.

### Variant detection

Using variant calling pipeline, 3.9 M and 4.7 M variants were initially detected in *C. arietinum* and *C. reticulatum* genome, respectively. Out of all variants, a total of 3.26 M SNPs were identified in *C. arietinum*, by contrast 3.93 M in *C. reticulatum* compared to the reference genome. In total, 35,329 and 44,331 InDels were identified in the species of *C. arietinum* and *C. reticulatum*, respectively. A total of 3387 InDels with 2 bp length was detected in *C. arietinum*, there was 4704 in *C. reticulatum.* Among 8091 InDels, 58 di-nucleotide regions that were polymorphic between two species were selected and used for primer design (Table 1).

**Table 1:** The primer sequences of the 58 SSR markers developed and used in this study.

Markers	Physical position	Forward primer (5′–3′)	Reverse primer (3′–5′)	Motif	Product length (bp)		
					*C. arietinum*	*C. reticulatum*	Reference genome (*C. arietinum*)
SSR1	19,011,134–19,011,210	CTTCCACGCGAGAGAAAAAC	TGGCCAATTTGAAAAGAAAA	CT	176	180	182
SSR2	55,753,401–55,753,477	TTGCCCTGATTTGAGATGTG	TTGGAAATTCAACCTACACAAAAA	TA	158	160	160
SSR3	19,011,133–19,011,211	CTTCCACGCGAGAGAAAAAC	TGGCCAATTTGAAAAGAAAA	CT	176	180	182
SSR4	889,577–889,653	TGCCAGTTTTTAACAGCATGA	CAGCATTATCTGCAAAAACAAA	AT	164	154	164
SSR5	994,011–994,087	TCCTTGTTTTAATTCCTCCATTG	TGAGACTCGACGCATTTAAGAA	TA	164	162	164
SSR6	1,322,967–1,323,044	TTCATGATGAGTGAATGGATGAG	AAATGGTGCACGTGTTTGTT	AT	167	157	167
SSR7	7,315,917–7,315,993	TGTTGCTGAGAAATTAAAAGAATGA	GCAACCAGACAAAACACGAG	TG	231	229	231
SSR8	13,721,274–13,721,350	CCAAATCCACTCCACCAGAT	ATGGGTCGAACAGGTGAAAC	AT	154	152	154
SSR9	21,156,335–21,156,411	CCATTGTTTTGACGGTGTTG	ATGGAGGAGTGGGTTTGACA	TA	185	183	185
SSR10	25,727,833–25,727,909	CGTTTGTTTGTTTTCATACACG	CACACAAATCTAGTCCCTTGAGA	AG	154	152	154
SSR11	32,040,766–32,040,842	TCTCACAGCAGTGGTCCTCTT	AATGTCAAATTGAAGCCACCT	CT	153	151	153
SSR12	47,883,120–47,883,196	CGCAGTGTGCAGAACAGAGA	TGAGAAAAGTGAAAAATGGAAGA	TC	164	162	164
SSR13	709,972–710,050	GAAGTTGAACACAGCCTCGTT	CAGAAAGAAGGACCAAAATTGTAA	TA	239	237	239
SSR14	3,754,394–3,754,472	GATCCTATGACGGCCAAGAT	CAATGTGGCACTAGAATAGCTG	TC	179	171	181
SSR15	4,508,947–4,509,025	GATGAATTGCAATGCCCACT	TGAGACCATACTTTTGCATCG	GA	152	148	154
SSR16	5,702,105–5,702,183	TTAGGCACACTTCCCATCAA	ACCCCACTTGTGATTTTTGC	AT	150	148	150
SSR17	5,706,723–5,706,801	CTCGCAAAAGAATGAATCACA	CACCAAATATATCAGAGTTCTCATGG	AT	150	148	150
SSR18	7,220,178–7,220,256	CCTGCATGCATCTCTCTCAT	TTGAACAGCATTGCCATCAT	AT	205	199	205
SSR19	11,523,580–11,523,658	AGCTCCGGACCTTTGAAATA	CCAGAATAGGTGGGGTTTCA	GA	163	161	163
SSR20	12,063,993–12,064,071	TCATCCTATTTTTGTGTATAAAATCGT	TGTTATTTTAGGATTTGTCAAGGTT	AG	229	227	229
SSR21	25,623,108–25,623,186	TGTTGGTGGCTCAACTATCAT	TGCGTTTTAGTTCAAACAACCTT	AG	184	182	184
SSR22	26,072,281–26,072,359	AGTGTGAATCAATCTGCTCTGA	TTAAACAAATCAAAGCATTGAAAA	GT	158	156	160
SSR23	30,200,833–30,200,911	TACAATTCAAAGCGGCACAA	CCCTTTGTGATATTTCTCGTGTT	TC	156	154	156
SSR24	34,024,156–34,024,235	TGATCACATTGCATCCATCTT	TGGAAATTGTGAGATTAAAACATAGAA	AT	179	177	179
SSR25	40,487,098–40,487,176	AAGCGAAGCGTACCTTTGAA	TCCTCTCCGCACTCTCTCTC	AG	139	137	139
SSR26	514,664–514,710	TTGAATCACCATCTGAAAAATCA	GGGCAAGCTCCAAGTACAGT	GA	311	309	311
SSR27	12,151,893–12,151,939	AACCTTTTTGAGATTGATTGAAGG	CCTTCAAATACACCAAAGGACA	TA	187	185	187
SSR28	13,690,483–13,690,529	TCCACAATGGAGATAAGAAAGC	TTGACTTGATTGGTTTGAGAGAA	CT	156	154	156
SSR29	20,110,687–20,110,733	TTTTGTATTGTCAATTTCGCATT	TTTCTCTCCCCCGTTACTCA	AG	172	170	172
SSR30	22,768,737–22,768,783	AAGTGATGGACACATGCAATCT	GGGATACGGATTTGGAGGGTA	AC	327	325	327
SSR31	30,049,660–30,049,706	CCACATGTTTCGTAGTGTTATCTCC	CTTGATTGAATTAAAGTTTGAAAAAG	AT	164	162	164
SSR32	3,762,999–3,763,051	AAACACAACAAAAGATCACATGG	TTTCAAAGAACCCCAACAGAA	AT	314	312	314
SSR33	4,183,287–4,183,339	TCCTTTTCCAAATTCCAATGA	GGAGCAGAGTGTGTGTGTGG	TC	153	151	153
SSR34	5,072,765–5,072,817	CAATTACATGTTAGATGACGTGCT	TGTTGCACACAAAAAGTTAGACG	TA	372	370	372
SSR35	7,847,913–7,847,965	TGGCCATTGGATTGGTTTAT	TGAAAACAAAAATGAACATGGAA	TA	130	128	130
SSR36	10,455,187–10,455,239	TCTTGTAAGTACGGTGGCAGTG	TATTGTTGCAAGAAATTGTCTCTTT	AC	150	146	150
SSR37	822,924–823,003	TGTCCAAGAACGACAATGTG	CGACTTAACATTAGCAATAGTCTTCAA	CA, AG	154	152	154
SSR38	7,250,162–7,250,240	AAATAGTCCATAAGCTTCACCATAC	TTGATTAATTACCACAACTTTATATGC	AT	152	150	152
SSR39	16,688,457–16,688,535	TGAGTGTTGTTGTTACCTTTTGC	CATCGACACAATTCCAAGGTT	GA	157	155	157
SSR40	20,328,085–20,328,163	AAAATTTAGAAAATGGGAGAAAACA	TGTGACATATGCATTTGCTCTTAC	GA	186	184	186
SSR41	24,316,422–24,316,500	AAAAACATCGAAACCAGCAAA	ACGTGTTCCCATTGGTTAGC	AG	341	339	341
SSR42	24,874,026–24,874,104	AGAAAAAGAGGACGAACAGAAA	TCTTTTGCTCCGTTGGATTT	AT	153	149	153
SSR43	27,746,809–27,746,887	GAATCGGAACTAAAACCGAAA	TCTCTCCCTCCCTCCCTCTA	GA	245	243	245
SSR44	29,532,448–29,532,526	TCAGAAATAGGAAAAGCAGTTTCA	CCTGAATGCCAAAATAAGGTTC	TA	205	203	205
SSR45	30,278,651–30,278,729	CCCGGTTTGTCGTGTCTATC	GAAAGGTGTTGGTTGGTGAT	TC	173	171	173
SSR46	30,896,220–30,896,298	TGGTTTTGTTACATTGCATCTG	TGCACATCACACACAAGGAA	TG	221	219	221
SSR47	36,420,939–36,421,017	TGCCATTGTTGAAAGCACAT	TCAAATGCTTCATTGCCATT	AT	317	313	317
SSR48	1,077,194–1,077,272	AACGTCCACAATGAGAAAAGC	GCCATTTCTTGCAAAGTTCA	TG	198	196	198
SSR49	207,413–207,491	TAACTTGGGCTTCGAGGAGA	AACTCTGCCGTATGCTTTCC	AG	155	151	155
SSR50	5,819,504–5,819,583	TGGTTGTTGCTATTTCAACCT	TGATTTGGGTCTCTTTTTGCTT	AT	200	198	200
SSR51	31,121,065–31,121,143	TTGTCTGAAGAATGCCACCTT	TTTGTGAAGCGTCACTCAGG	AT	144	140	144
SSR52	31,568,270–31,568,348	TCAACCCACGTGCTTTTGTa	CCGGTCAATATTTTGCGAGT	AT	196	194	196
SSR53	33,184,427–33,184,505	AAAACATTCTGCAATTTTGTTTTA	TCTCGTTGTTCAAACCCAAAC	AT	162	158	162
SSR54	33,645,811–33,645,889	TGCCTTTGTACTCTTCTATATTTGG	CAAATTGTTTGCCTTTTGTTTG	AT	230	228	230
SSR55	36,678,457–36,678,535	GTTCGTCATACGATAAGAAGAGAAA	TATAGCGTCGGTTGTCAATTTTT	AT	150	146	150
SSR56	37,082,804–37,082,882	GCACCCACACCTGCTAAGAG	TCCCAAGAACGTCTTTCACC	TC	162	152	162
SSR57	4,181,347–4,181,425	AAGTCCTAATATTGGGCTGTTTAGA	TATGCATGCAGAAACACACG	AT	152	150	152
SSR58	2,837,886–2,837,964	GGTGTGATGTGTGGCAGAGA	GCCCGGAAATACAGGGATAC	AG	186	182	186

### SSR validation in RIL population

Designed primer pairs were used for validation in 30 chickpea genotypes of F_6_ population obtained from an interspecific cross between *C. arietinum* and *C. reticulatum.* Out of SSR31 and SSR32, all primers were successfully amplified. The obtained PCR products were loaded on a polyacrylamide gel, and allele sizes were determined by comparing with *C. arietinum* and *C. reticulatum*. The difference of allele sizes was also confirmed in the gel. It was seen that all 30 genotypes carried one of the alleles which the parents had. While SSR5 and SSR10 produced suitable alleles in 30 RIL genotypes for 2-nucleotide polymorphism between female and male parents, SSR14 primer produced suitable alleles for 8-nucleotide polymorphism and SSR18 primer for 6-nucleotide polymorphism between *C. arietinum* and *C. reticulatum* (Table 1).

Chi-square (χ^2^) values were calculated for each marker to test the fit of the markers in 30 genotypes representing the RIL population to the expected 1:1 expression ratio. Markers deviating from expected Mendelian ratios were determined by chi-square analysis (Table 2). According to the results, it was determined that the markers were suitable for 1:1 expansion ratio, since the calculated p values for all markers except SSR20 were greater than 0.05.

**Table 2:** Chi-square (χ^2^) values for each marker to test the fit of the markers in the RIL population to the expected 1:1 expression ratio.

Markers	Allele size (bp)		Chi-square values	*P* values
	*C. arietinum*	*C. reticulatum*		
SSR1	176	180	0.154	0.695
SSR2	158	160	1.000	0.317
SSR3	176	180	3.846	0.050
SSR4	164	154	0.034	0.853
SSR5	164	162	0.133	0.715
SSR6	167	157	0.037	0.847
SSR10	154	152	1.815	0.178
SSR11	153	151	2.133	0.144
SSR14	179	171	0.143	0.705
SSR15	152	148	0.040	0.841
SSR16	150	148	0.000	1.000
SSR17	150	148	0.040	0.841
SSR18	205	199	0.000	1.000
SSR19	163	161	0.000	1.000
SSR20	229	227	26.133	0.000
SSR21	184	182	2.793	0.095
SSR22	158	156	0.926	0.336
SSR23	156	154	2.286	0.131
SSR24	179	177	0.048	0.827
SSR26	311	309	0.310	0.577
SSR27	187	185	0.167	0.683
SSR29	172	170	0.615	0.433
SSR30	327	325	0.333	0.564
SSR37	154	152	0.310	0.577
SSR38	152	150	0.310	0.577
SSR39	157	155	0.143	0.705
SSR40	186	184	0.034	0.853
SSR41	341	339	0.142	0.705
SSR42	153	149	0.310	0.577
SSR43	245	243	0.533	0.465
SSR45	173	171	0.333	0.564
SSR46	221	219	0.143	0.705
SSR47	317	313	0.926	0.336
SSR51	144	140	0.533	0.465

### SSR diversity in cultivated and wild populations

**Figure 1:**
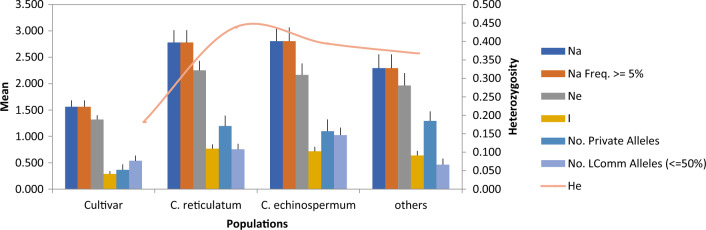
Allelic patterns and gene diversity across cultivated and wild populations. The figure shows comparison for number of alleles (Na), Number of alleles with frequency more than or equal to 5%, Number of effective alleles (Ne) and Number of private alleles, etc.

For genetic diversity analysis, 30 genotypes obtained from cultivated and wild species were tested in polyacrylamide gel, bands were scored according to allele sizes. As a result of the analysis, a total of 244 alleles belonging to 41 different SSR loci were determined in 30 chickpea genotypes (Table 3). At the population level, allelic diversity in cultivated and wild populations was shown in Fig. 1. Total allele distribution was 63 in cultivars and 311 in wild genotypes. While a total of 110 alleles were determined in the genotypes of the *C. reticulatum*, 112 alleles were observed in the genotypes of the *C. echinospermum*. 89 alleles were determined in the population from distantly related wild species. The mean number of alleles (Na) for 30 genotypes was 2.36 (Table 3). The highest number of alleles was obtained from the primers SSR3, SSR58 and SSR39 (Table 3). The number of effective alleles (Ne) varied between 0.75 and 3.74. Nei's^54^ observed (Ho) and expected (He) heterozygosity values were calculated as 0.08 and 0.34, respectively. The mean of polymorphism information content (PIC) was measured as 0.73 (Table 3). The highest PIC value was observed at the SSR21 (0.90) loci, followed by the SSR56 (0.88), SSR54 (0.86), SSR4 (0.85), SSR7 (0.83) and SSR34 (0.83) loci. The lowest PIC value was found in the SSR9 (0.51) locus (Table 3).

**Table 3:** Summary of genetic diversity statistics for 30 chickpea genotypes.

Markers/loci	N	Na	Ne	I	Ho	He	uHe	F	PIC
SSR2	7.000	3.750	2.766	1.003	0.083	0.525	0.566	0.675	0.826
SSR3	7.250	5.250	3.743	1.382	0.163	0.665	0.718	0.788	0.781
SSR4	7.250	3.750	2.986	1.122	0.071	0.617	0.663	0.908	0.854
SSR5	7.250	2.500	2.157	0.726	0.167	0.432	0.466	0.680	0.645
SSR6	6.750	1.250	1.038	0.064	0.036	0.033	0.036	-0.077	0.637
SSR7	6.250	3.750	3.340	1.101	0.077	0.586	0.643	0.894	0.833
SSR8	6.750	2.750	1.783	0.716	0.100	0.418	0.454	0.828	0.749
SSR9	5.750	1.500	1.320	0.267	0.000	0.180	0.201	1.000	0.51
SSR10	5.500	1.000	0.938	0.155	0.031	0.107	0.115	0.709	0.664
SSR11	6.250	3.500	3.056	1.128	0.154	0.645	0.702	0.740	0.816
SSR12	6.000	2.250	1.900	0.624	0.000	0.385	0.425	1.000	0.689
SSR13	5.000	1.750	1.454	0.372	0.071	0.237	0.257	0.682	0.826
SSR16	7.000	2.500	1.819	0.595	0.217	0.335	0.366	0.243	0.608
SSR17	4.750	2.000	1.497	0.540	0.550	0.330	0.358	-0.559	0.817
SSR18	5.250	3.250	2.983	0.974	0.000	0.523	0.573	1.000	0.690
SSR19	6.750	1.500	1.322	0.290	0.000	0.196	0.210	1.000	0.731
SSR21	5.250	3.250	2.644	0.937	0.000	0.508	0.554	1.000	0.898
SSR25	5.750	2.000	1.753	0.552	0.167	0.369	0.432	0.610	0.736
SSR28	5.250	1.500	1.431	0.326	0.050	0.230	0.283	0.762	0.717
SSR33	6.750	2.000	1.766	0.537	0.000	0.344	0.376	1.000	0.615
SSR34	7.000	2.250	2.083	0.594	0.000	0.340	0.369	1.000	0.827
SSR35	5.250	1.500	1.204	0.199	0.000	0.112	0.121	1.000	0.796
SSR36	7.000	1.750	1.462	0.406	0.094	0.271	0.297	0.534	0.545
SSR37	5.750	2.000	1.637	0.495	0.063	0.305	0.344	0.619	0.599
SSR38	3.000	0.750	0.750	0.173	0.000	0.125	0.143	1.000	0.814
SSR39	7.000	4.000	3.480	1.141	0.155	0.596	0.644	0.807	0.773
SSR42	6.000	1.500	1.331	0.276	0.000	0.186	0.233	1.000	0.645
SSR43	7.500	2.000	1.683	0.512	0.063	0.325	0.350	0.619	0.717
SSR44	6.750	2.250	1.920	0.588	0.031	0.349	0.386	0.644	0.615
SSR45	6.750	2.750	2.140	0.700	0.077	0.381	0.413	0.569	0.65
SSR46	6.750	2.000	1.561	0.495	0.125	0.318	0.347	0.590	0.78
SSR49	6.000	2.750	2.541	0.807	0.000	0.445	0.475	1.000	0.753
SSR50	6.750	2.000	1.591	0.440	0.250	0.266	0.283	0.137	0.788
SSR51	6.500	2.750	1.929	0.583	0.083	0.292	0.314	0.600	0.77
SSR52	5.500	1.250	0.992	0.244	0.063	0.157	0.168	0.429	0.692
SSR53	6.000	1.750	1.233	0.394	0.031	0.229	0.244	0.805	0.736
SSR54	6.250	2.250	1.705	0.591	0.125	0.364	0.397	0.429	0.864
SSR55	6.250	1.750	1.487	0.354	0.000	0.211	0.225	1.000	0.754
SSR56	5.750	3.250	2.345	0.952	0.063	0.546	0.599	0.846	0.876
SSR57	5.750	1.000	0.831	0.103	0.000	0.061	0.066	1.000	0.656
SSR58	7.500	4.250	3.359	1.205	0.125	0.618	0.664	0.832	0.825
Mean	6.213	2.360	1.926	0.602	0.080	0.345	0.378	0.715	0.735

Number of alleles (Na), number of effective alleles (Ne), Shannon diversity index (I), Expected heterozygosity (He), Unexpected heterozygosity (uHe), Observed heterozygosity (Ho), Wright’s fixation index (F), Polymorphic information content (PIC).

**Figure 2:**
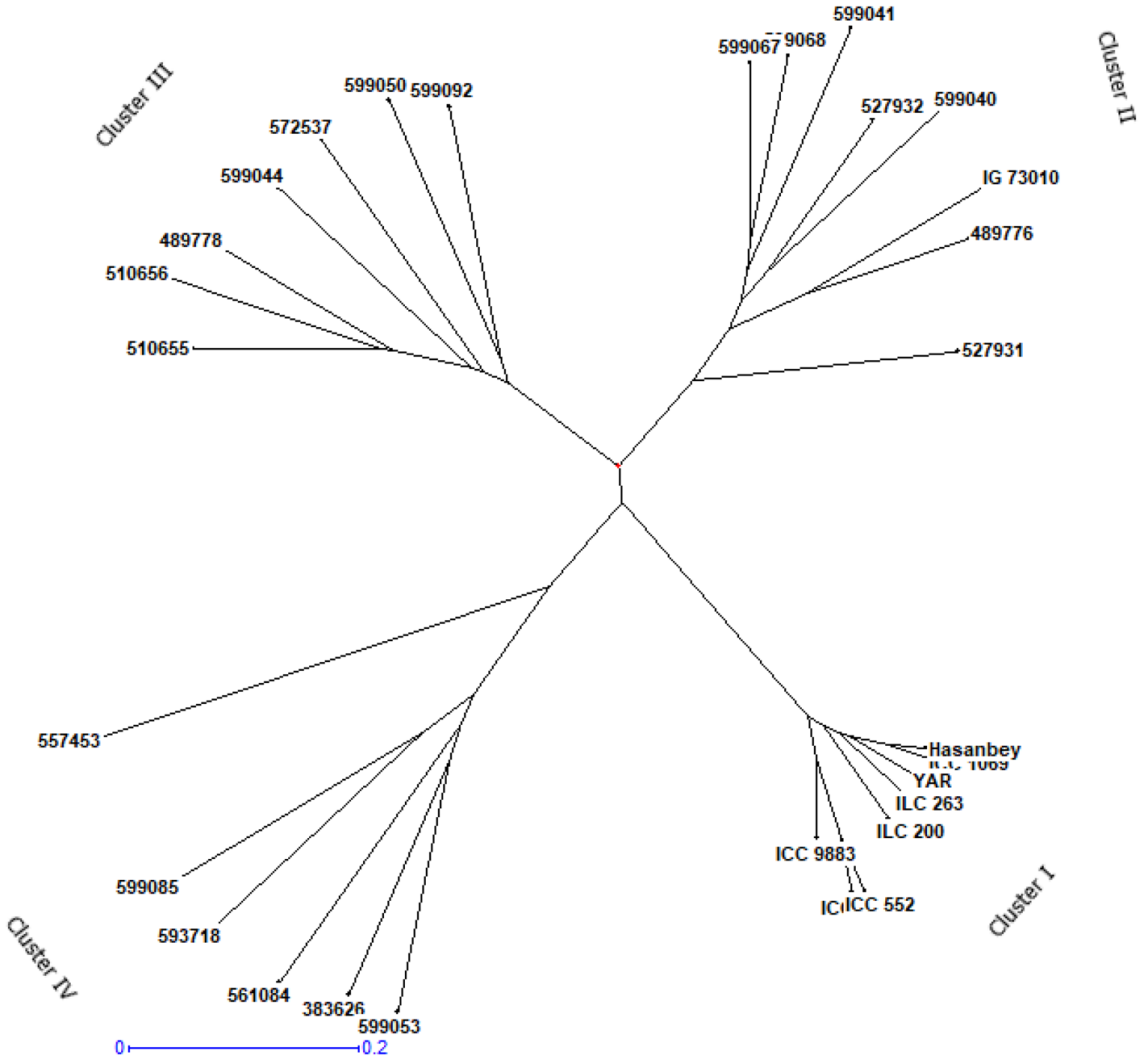
UPGMA based dendrogram generated using SSR markers and 30 wild and cultivated chickpea genotypes.

Phylogenetic tree consisting of 30 chickpea genotypes was constructed based on the UPGMA clustering method with newly developed SSRs (Fig. 2). The chickpea genotypes were divided into four clusters, indicating clear separation between wild and cultivated species. Cluster I contained cultivated chickpeas including four kabuli and four desi chickpeas. Cluster II, III and IV consist of wild chickpea species, each representing *C. echinospermum, C. reticulatum* and other wild chickpea species, respectively.

**Figure 3:**
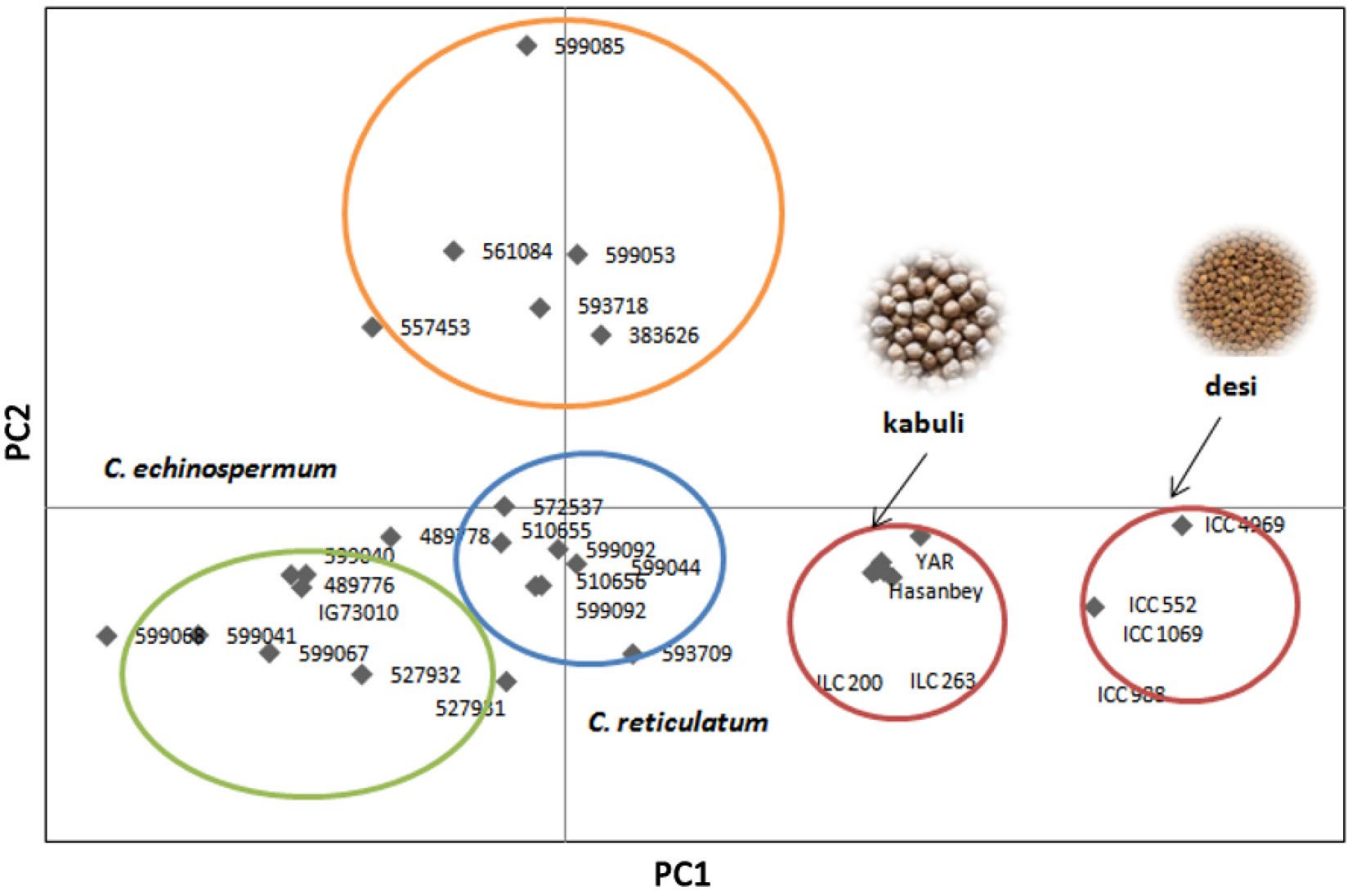
Principal coordinate analysis (PCoA) of the 30 chickpea genotypes genotypes with SSR markers.

The PCoA analysis confirmed the clusters of the phylogenetic tree (Fig. 3). Cultivated and wild genotypes did not cluster together. The two informative components explained 92.36% of the cumulative variance, PC1 and PC2 shared 53.72% and 38.64% variation, respectively.

## Discussion

### Using NGS technology is an effective tool for the identification of SSR markers

SSRs are valuable genetic markers due to their co-dominant inheritance, multi-allelic and reproducible nature^55^. In chickpea, large numbers of SSR markers have been identified and widely used for genetic diversity analysis, gene/QTL mapping, construction of linkage map, marker assisted selection (MAS)^33,56–59^. However, validation and selection of informative markers from such huge numbers of markers that show polymorphism in chickpea, is an excessive effort. In addition, the narrow genetic base in chickpea may can restrict use of the identified markers in genotyping studies because of their low intra-specific polymorphism among chickpea genotypes^23,30^. The NGS technologies have caused impressive advances in sequencing which creates high-throughput sequences to transform genotyping and plant breeding. It provides opportunities to perform high-throughput SSR identification. In present study, we developed genome-wide SSR markers from cultivated and wild chickpea genotypes. SSR marker development from genomic data has been reported for various crops such as sesame^60^, red clover^61^, peanut^62^, sweet potato^63^, faba bean^64^, lentil^65^.

### Distribution of variants in *C. arietinum* and *C. reticulatum* genome

As a result of alignment to the reference genome of chickpea, a total of 3.26 M SNPs were identified in *C. arietinum*, by contrast 3.93 M in *C. reticulatum*. Previously, 51,632 SNPs were reported by 454 transcriptome sequencing of *C. arietinum* and *C. reticulatum* genotypes^35^. In addition, couple hundreds of SNPs were also studied using Solexa ⁄ Illumina sequencing, targeted amplicon sequencing, mining of expressed sequence tag libraries and sequencing of candidate genes^30,66,67^.

### Validation and polymorphic potential of SSRs

The utilization of genetic diversity in chickpea genetic resources is very important in order to utilize collections and improve breeding studies. Genetic diversity analysis in chickpea was previously performed using RAPD^18^, AFLP^68^, STMS^69^, SSRs^70,71^. In this study, the effectiveness of the developed markers was evaluated in 30 chickpea genotypes obtained from cultivated and wild species as well as 30 chickpea genotypes of F_6_ population obtained from an interspecific cross between *C. arietinum* and *C. reticulatum.* The markers were effective for detection of a total of 244 alleles (Na). The mean of number of alleles (2.36) observed in this study are within the ranges revealed by various previous studies. For instance, the use of 33 SSR markers identifed a total of 111 alleles with an average of 3.7 alleles per locus in 155 chickpea genotypes^72^. Similarly, 27 SSRs were used to study genetic diversity in 50 chickpea accessions which reported a total of 81 alleles with an average of 3.0 alleles/locus^73^. In the present study, heterozygosity was detected in genotypes that ranged from 0.03 to 0.66 with mean of 0.34, which is similar to previous studies reported previously by Upadhyaya et al.^74^ and Hajibarat et al.^75^. Genetic diversity analysis showed that the average PIC value of SSR markers was 0.73, higher than PIC value of the SNPs^76^, STMS^77,78^, AFLP^20^ and SilicoDArT^79^ markers used to identify genetic variation in chickpea. Botstein et al.^80^ reported the PIC values of markers as highly informative (≥ 0.5), reasonably informative (0.50–0.25), or least informative (≤ 0.25). Our average PIC value (0.73) thus shows that the developed markers identified here are highly informative and greatly sufficient for showing relationships among genotypes, according to Meszaros et al.^81^. The principal coordinate analysis clearly separated the whole population into four clusters, and wild and cultivated types in seperate clusters. Results from the present study are consistant with the previous studies^71,82^ the grouping followed a clear pattern between cultivated chickpea and the wild species. It is also clear as the wild progenitor, *Cicer reticulatum* showed close proximity with the cultivated chickpea. The other close connection was seen between *C. reticultum* and *C. echinospermum*. It can be supposed from this study that cluster analysis shows the effectiveness of the designed markers.

The results of the present study revealed the success of SSR identification and marker development in chickpea using NGS genome data. The developed SSR markers were applied successfully for illuminating genetic diversity among cultivated and wild chickpea populations as well as validation in F_6_ population obtained from an interspecific cross between *C. arietinum* and *C. reticulatum.* Therefore, newly developed 58 SSR markers are potentially useful for genetic studies of chickpea.

In conclusion, NGS strategy led to the discovery of a large number of microsatellites markers, providing thousands of SSRs for validation in chickpea. These new SSRs will become significant molecular tools for chickpea genetic breeding programs. Later, these markers could be integrated in genetic maps to be utilized in MAS.

## Materials and methods

### Plant material

*C. arietinum* L.*,* CA 2969 and *C. reticulatum* Ladiz.*,* AWC 602 were used as a genetic material for WGRS analysis. CA 2969 and AWC 602 chickpea genotypes were registered by USDA-ARS and Akdeniz University, Department of Field Crops, respectively. The important traits for these genotypes were given in Table 4. Developed SSRs were validated in 30 chickpea lines from a RIL population earlier developed by Sari et al.^83^ and derived from an interspecific cross between CA 2969 and AWC 602. The markers were also used to assess the genetic diversity of cultivated and wild chickpea accessions including eight accessions of *C. arietinum* (four kabuli and four desi chickpeas), eight accessions of *C. reticulatum*, eight accessions of *C. echinospermum* P.H. Davis and six accessions of *C. anatolicum* Alef., *C. canariense* A. Santos & G.P. Lewis*, C. microphyllum* Benth., *C. multijugum* Maesen, *C. oxyodon* Boiss. & Hohen. and *C. songaricum* Steph ex DC. (Table 5). Seed samples of ICARDA and USDA are available directly from ICARDA (https://www.icarda.org/) and USDA (https://www.usda.gov/). The procurement of seeds of all cultivated and wild genotypes used in the present study complies with relevant institutional, national, and international guidelines and legislation.

**Table 4:** Important morphological and the specific-known traits of the parents used for WGRS analysis (*Chrigui et al.^15^).

Traits	Species	
	*C. arietinum* (CA 2969)	*C. reticulatum* (AWC 602)
Kabuli/desi or wild	Kabuli	Wild
Flower color	White	Purple
Pod per axis	2	1
Seed color	Cream	Brown
100-seed weight (g)	34	21
Cold tolerance	Susceptible	Tolerant
Resistance to pulse beetle	Susceptible	Resistant
Resistance to leafminer*	Susceptible	Resistant

**Table 5:** Cultivated and wild *Cicer* species.

No.	Species	Genebank no	Kabuli/desi/wild	Annual/perennial	Genebank/institute	Origin
1	*C. arietinum*	Hasanbey	Kabuli	Annual	EMARI	Turkey
2	*C. arietinum*	YAR	Kabuli	Annual	Akdeniz University	Turkey
3	*C. arietinum*	ILC 200	Kabuli	Annual	ICARDA	Turkey
4	*C. arietinum*	ILC 263	Kabuli	Annual	ICARDA	Turkey
5	*C. arietinum*	ICC 4969	Desi	Annual	ICRISAT	Turkey
6	*C. arietinum*	ICC 552	Desi	Annual	ICRISAT	Turkey
7	*C. arietinum*	ICC 988	Desi	Annual	ICRISAT	Turkey
8	*C. arietinum*	ICC 1069	Desi	Annual	ICRISAT	Turkey
9	*C. reticulatum*	593709	Wild	Annual	USDA	Turkey
10	*C. reticulatum*	510656	Wild	Annual	USDA	Turkey
11	*C. reticulatum*	599092	Wild	Annual	USDA	Turkey
12	*C. reticulatum*	599050	Wild	Annual	USDA	Turkey
13	*C. reticulatum*	599044	Wild	Annual	USDA	Turkey
14	*C. reticulatum*	510655	Wild	Annual	USDA	Turkey
15	*C. reticulatum*	572537	Wild	Annual	USDA	Turkey
16	*C. reticulatum*	489778	Wild	Annual	USDA	Turkey
17	*C. echinospermum*	599040	Wild	Annual	USDA	Turkey
18	*C. echinospermum*	599041	Wild	Annual	USDA	Turkey
19	*C. echinospermum*	599068	Wild	Annual	USDA	Turkey
20	*C. echinospermum*	527932	Wild	Annual	USDA	Turkey
21	*C. echinospermum*	489776	Wild	Annual	USDA	Turkey
22	*C. echinospermum*	599067	Wild	Annual	USDA	Turkey
23	*C. echinospermum*	527931	Wild	Annual	USDA	Turkey
24	*C. echinospermum*	IG 73010	Wild	Annual	ICARDA	Turkey
25	*C. canariense*	557453	Wild	Perennial	USDA	Spain
26	*C. anatolicum*	383626	Wild	Perennial	USDA	Turkey
27	*C. multijugum*	599085	Wild	Perennial	USDA	Uzbekistan
28	*C. microphyllum*	593718	Wild	Perennial	USDA	India
29	*C. oxyodon*	561084	Wild	Perennial	USDA	Turkey
30	*C. songaricum*	599053	Wild	Perennial	USDA	Uzbekistan

Eastern Mediterranean Agricultural Research Institute (EMARI), The International Center for Agricultural Research in the Dry Areas (ICARDA), The International Crops Research Institute for the Semi-Arid Tropics (ICRISAT), The United States Department of Agriculture (USDA).

### Experimental area

Plants belonging the parents (CA 2969 and AWC 602) and 30 cultivated and wild chickpea accessions were grown in separate pods in a greenhouse at the Faculty of Agriculture, Akdeniz University, Antalya, Turkey (30°38′E, 36°53′N, 33 m above sea level) for genomic DNA extraction.

### DNA extraction

DNA extraction process was carried out at Plant Molecular Biology Laboratuary, the Faculty of Agriculture, Akdeniz University, Antalya, Turkey. Genomic DNA was extracted from 3 week-old young leaves of plants individually using the CTAB method as described by Doyle and Doyle^84^ with minor adjustments such as extra chloroform-isoamyl alcohol and 70% ethanol cleaning steps. DNA quality and quantity of each sample were estimated by electrophoresis on 1% agarose gels, and the amount was fixed to 100 ng/μL using lambda DNA as a reference.

### Library preparation and sequencing

The genomic data from *C. arietinum* and *C. reticulatum* was used for construction of a HiSeq sequencing library using TruSeq DNA sample Prep kit LT, (set A) FC-121-2001 (Illumina, San Diego, CA, USA) according to manufacturer’s protocol. A reduced representative genomic library with a target insert size of about 350 bp were sequenced on Illumina Hiseq X to generate 150-bp paired-end reads at Macrogen Inc., (Macrogen, Seoul, Korea). WGRS data of two available genotypes were deposited into the National Center for Biotechnology Information (NCBI) Sequence-Read Archive (SRA) database.

The raw data were demultiplexed using Je V1.2^85^, a quality control was performed for FASTQ Sanger files using fastp^86^, and reads with a Phred quality score below 15 were trimmed^87^. The cleaned data were aligned with kabuli reference genome 1.0^1^ using Bowtie 2 with default parameters^88^ in the Galaxy software (www.usegalaxy.org). The created BAM files (*.bam) were analyzed using Freebayes (Galaxy Version 1.1.0.46-0)^89^, with simple diploid calling and filtering, and a minimum of 20 × coverage for variant detection. The obtained variant files were filtered using VCFfilter (Galaxy Version 1.0.0) and SNPs were chosen. Insertions and deletions from individual (*.vcf) files were later merged into a single VCF file using VCF genotypes (Galaxy Version 1.0.0).

The combined variant file was processed using Microsoft Excel to eliminate duplicated regions and organize the SSRs according to their sizes. SSR regions which have 2 bp long and polymorphic between parents were checked using the Integrated Genome Browser V9.1.4.

### Primer design

For designing the primer pairs from the flanking sequences of identified SSRs, Primer3 software^90,91^ was used with the parameters as follows: primer length of 18–27 nucleotides, melting temperatures of 55–65 °C, GC content of 30–70%, and predicted PCR products of 100–300 bp in length. The primer pairs were later controlled for possible duplication of sequences in the genome using IGB software.

The PCR reactions were performed using the M13 tailing PCR procedure^92^. The forward primers were tailed by adding an M13 sequence labeled with IRDye to the 5′ end. The following PCR protocol was applied: 95 °C initial denaturation for 5 min, 30 cycles at 95 °C for 30 s, annealing temperature 60 °C for 30 s, 72 °C for 1 min, followed by 9 cycles of 95 °C for 30 s, 55 °C for 30 s, 72 °C for 1 min, and then a final extension of 10 min at 72 °C. PCR products were loaded onto 8% denatured polyacrylamide gel and separated by 4300 DNA analyzer (LI-COR, Inc., Lincoln, Nebraska, USA). 1 kb size marker was used to score markers as 1 or 0 for the presence and absence of alleles.

### Statistical analyses

RIL data was analyzed using MINITAB 19 software. A Chi square (χ^2^) test was used to assess goodness of fit to the observed segregation ratios followed 3:1 ratio in the RIL population.

### Genetic diversity and phylogeny analysis

Genetic diversity parameters such as number of alleles (Na), number of effective alleles (Ne), Shannon diversity index (I), expected heterozygosity (He), unexpected heterozygosity (uHe), observed heterozygosity (Ho) and Wright’s fixation index (F) were shown using GenAlEx 6.5^93^. The phylogenetic tree was constructed in DARwin ver 5.0 software^94^ using the unweighted pair group method with arithmetic mean (UPGMA)^95^ clustering method and modified in FigTree v1.4.4 (http://tree.bio.ed.ac.uk/software/figtree). Principal coordinate analysis (PCoA) was performed with GenAlEx 6.5 to evaluate the genetic relationships between populations. The Excel microsatellite toolkit^96^ was used to measure polymorphism.

## Data Availability

The datasets generated and analysed during the current study are available in the National Center for Biotechnology Information (NCBI) Sequence-Read Archive (SRA) database with the accession number of PRJNA926661.
